# Multivariate Associations Between R-PAS Variables and Suicidal Ideation and Behaviors in Help-Seeking Adolescents

**DOI:** 10.3390/brainsci16050519

**Published:** 2026-05-13

**Authors:** Marzia Di Girolamo, Roberta Invernizzi, Paola Galli, Irene Orlandi, Luciano Giromini, Donald J. Viglione, Renato Borgatti, Martina Maria Mensi, Marika Orlandi

**Affiliations:** 1 Department of Psychology, University of Turin, 10124 Turin, Italy; marzia.digirolamo@unito.it (M.D.G.);; 2 Child Neurology and Psychiatry Unit, ASST Lecco, 23900 Lecco, Italy; 3 Department of Medicine and Surgery, University of Milano-Bicocca, 20126 Milan, Italy; 4 Clinical Psychology PhD Program, California School of Professional Psychology, San Diego, CA 92193, USA; 5 Department of Brain and Behavioral Sciences, University of Pavia, 27100 Pavia, Italymarika.orlandi@mondino.it (M.O.); 6 Child Neurology and Psychiatry Unit, IRCCS Mondino Foundation, 27100 Pavia, Italy

**Keywords:** cognitive complexity, morbid content, Rorschach Performance Assessment System, perceptual rigidity, suicidality

## Abstract

**Highlights:**

**What are the main findings?**

- Elastic net models identified MOR as the most consistent, with LSO-Cmplx and VFD as additional, multivariate correlates of suicidal ideation severity and lifetime suicidal behavior in help-seeking adolescents.
- The observed associations were modest in magnitude and only partially stable, indicating limited explanatory power and supporting an exploratory interpretation.

**What are the implications of the main findings?**

- The findings describe a multivariate pattern involving dysphoric ideational content, greater effort in scanning the environment, and avoidance-related indicators, without supporting predictive or diagnostic use.
- R-PAS variables may contribute descriptive information on cognitive–affective functioning within a comprehensive assessment, prompting the consideration of monitoring possible suicidal thoughts and behaviors.

**Abstract:**

**Background/Objectives:** Suicidal ideation and behavior in adolescence remain difficult to assess due to their multifactorial and fluctuating nature. Performance-based measures may provide additional information on psychological correlates associated with suicidality beyond self-report and interview data. **Methods**: This study examined multivariate associations between RorschachPerformance Assessment System (R-PAS) variables and suicidality in a clinical sample of 153 help-seeking adolescents. Elastic net penalized regression models were estimated to evaluate joint patterns of R-PAS variables derived from the Suicide Concern Composite (SC-Comp) and selected developmentally relevant indices, controlling for age, sex at birth, sociodemographic and clinical factors. Suicidal ideation severity and lifetime suicidal behavior were assessed using the Columbia–Suicide Severity Rating Scale (C-SSRS). **Results**: For both outcomes, morbid content (MOR) and Location, Space, and Object Qualities-Complexity (LSO-Cmplx) emerged as consistent multivariate correlates, with vigilance–avoidance (VFD) contributing only in the model for suicidal ideation severity. Model performance was modest (R^2^ = 0.09), indicating limited explanatory power. The findings do not support clinical or predictive use of R-PAS variables for suicidal ideation and behaviors, but indicate a small set of reproducible multivariate associations within this sample. **Conclusions**: The results suggest that certain R-PAS variables show weak but consistent associations with suicidality in a help-seeking adolescent sample. Given the cross-sectional design and modest explanatory power, the findings should be interpreted as exploratory and hypothesis-generating, and further studies are needed to clarify the robustness and meaning of these associations.

## 1. Introduction

Suicide is the second leading cause of death for people aged 15–24 [1] and a severe public health issue. Among adolescents (15–18 years old), the global suicide rate is around 6.2 per 100,000 annually, with males at higher risk, though the gender gap is smaller in adolescence than adulthood [2].

From a developmental perspective, suicidal thoughts and attempts tend to increase in early adolescence, peak around age 14–15, and then decline [3]. Dramatically, about one-third of adolescents with suicidal thoughts attempt suicide within a year, and the lifetime prevalence of suicidal ideation is 12.1% [4].

Despite the high prevalence and clinical relevance of suicidal phenomena in adolescence, the prediction of suicidal thoughts and behaviors remains limited. Meta-analytic findings indicate that commonly used risk assessment approaches perform only marginally better than chance (i.e., AUCs of 0.56–0.58) [5].

This limited predictive accuracy likely reflects the multifactorial and transient nature of suicidal thoughts and behaviors, which fluctuate over time and are influenced by interacting psychological, interpersonal, and environmental factors [6]. Accordingly, contemporary research has increasingly emphasized the limitations of purely predictive models and the need to better understand the psychological processes associated with suicidal experiences.

Consistent with this complexity, studies have identified both risk [7,8,9,10,11] and protective factors against suicidal ideation and attempts [12,13], but there is no consensus regarding optimal assessment strategies [14], with some studies aiming to predict eventual suicide attempts, while others focus on imminent ones [14,15].

Additional variability arises from methodological differences, such as reliance on self-report and informant-report measures versus clinician-administered tools, further complicating the comparability and integration of the findings.

Within this context, performance-based assessment methods are particularly well-suited to capturing implicit processes regulated at the unconscious level, and have been proposed as a complementary approach for investigating underlying psychological processes that may not be fully captured through self-report instruments. The Rorschach Performance Assessment System (R-PAS) [16] is a contemporary system for administering, coding and interpreting Rorschach. Empirical studies have reported evidence of validity and reliability [17,18,19], although the use of performance-based measures remains subject to ongoing debate regarding their interpretive scope and clinical utility.

There is currently a lack of systematic research on the specific validity of the R-PAS Suicide Concern Composite (SC-Comp) with adolescent samples. The empirical basis of the SC-Comp with adolescents focuses on reliability data, which is sufficiently good [20,21]. However, criterion validity remains an open research gap.

A recent narrative review by Zare et al. [22] covered studies from 1960 to 2021 and synthesized three main methodological approaches: single-item indicators, multi-item configurational approaches, and suicide constellation indices. Among single-marker indicators, responses regarding color-shading emerged as the variable most consistently supported across all studies. The Suicide Constellation (S-CON), developed within the Comprehensive System (CS), remains the most established composite index. However, the same review highlighted that no proposed indicator, whether single-sign or composite, has achieved consistent replication across independent samples. These findings align with the broader meta-analytic evidence on suicide risk assessment [5].

While the S-CON (CS) remains the most widely documented index to date, the SC-Comp (R-PAS) has been designed to refine its clinical application and statistical stability.

Among R-PAS-derived indicators, the SC-Comp represents a dimensional index intended to capture psychological features associated with suicide risk derived from the CS’s S-CON [23].

In light of that, the present study adopts an exploratory, cross-sectional approach to examine whether and, eventually, how multivariate patterns of SC-Comp components and developmentally relevant R-PAS indices are associated with the severity of suicidal ideation and the presence of suicidal behavior in a sample of help-seeking adolescents, a population less represented in prior works. Rather than aiming to establish predictive utility, this study seeks to investigate whether performance-based indicators capture statistically detectable patterns of association with clinically assessed suicidal thoughts and behaviors.

## 2. Materials and Methods

### 2.1. Design

This clinical cross-sectional study was conducted to examine contemporaneous associations between R-PAS variables and suicidal outcomes, but does not allow for causal or temporal inferences regarding these relationships. We followed the Declaration of Helsinki (1964) and its later amendments, and this study received approval from the Policlinico San Matteo Ethics Committee in Pavia (P-20200055757). This study was conducted in accordance with the Reporting of Studies Conducted using Observational Routinely collected health Data (RECORD) guidelines (Table S1). The participants’ parents/caregivers gave their written informed consent to participate in this study and data processing and were free to withdraw their participation in this study at any time. The dataset generated and analyzed during the current study was de-identified, pseudonymized and is available in the Zenodo repository [24].

### 2.2. Participants

**Figure 1:**
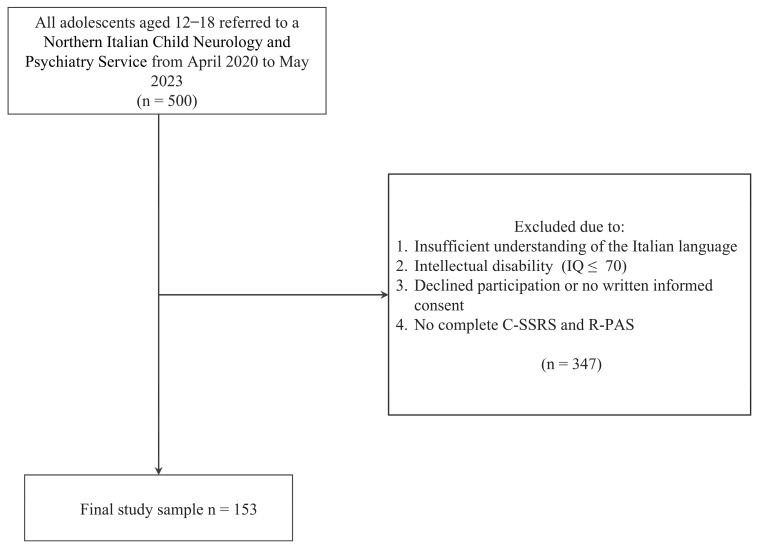
Study population flowchart.

A total of 153 adolescents, male (22) and female (131), aged 12 to 18 years (M = 15.1, SD = 1.44), were recruited from April 2020 to May 2023. Participants were help-seeking adolescents with severe psychiatric symptoms recruited from Northern Italian Child Neurology and Psychiatry Services. We proposed this study to all inpatient and outpatient adolescent patients, excluding those presenting with intellectual disability, as assessed through the Wechsler Intelligence Scale for Children (WISC-IV) [25,26] or the Wechsler Adult Intelligence Scale (WAIS-IV) [27,28], or those with insufficient understanding of the Italian language. We also excluded those who refused to participate or did not provide written informed consent, and those with missing data on the primary study instruments. Figure 1 shows the study population flowchart. The sample included adolescents presenting with a range of psychiatric conditions typically observed in help-seeking populations, especially mood, anxiety, psychotic, and eating disorders.

### 2.3. Procedures

Assessments were conducted over a period of approximately 3 to 10 days, depending on clinical scheduling, and required up to 2 h of direct administration. Participation was voluntary, and refusal to participate did not affect access to clinical care or treatment planning. All assessments were conducted by trained clinicians or psychologists and discussed in clinical supervision to support consistency in administration and scoring. All subjects were assessed at first admission to the facility through clinical interviews, with sociodemographic and anamnestic data collection. Then, a trained clinician or psychologist administered:

Columbia-Suicide Severity Rating Scale-Children Baseline Screening (C-SSRS) [29]: a semi-structured clinical interview that assesses suicidal ideation severity and frequency of the most severe ideation reported, lifetime and during the past six months. Suicidal ideation severity is a continuous variable that represents the highest level of suicidal ideation endorsed in the first part of the C-SSRS on 5 questions with a dichotomous response format (present/absent) (1 = wish to be dead, 2 = non-specific active suicidal thoughts, 3 = active suicidal ideation with any method but without a specific plan or the intent to act, 4 = active suicidal ideation with some intent to act but without a specific plan, 5 = active suicidal ideation with a detailed plan and intention to die). In the second part, it assesses the lifetime history of suicidal behaviors (actual attempts, interrupted attempts, where someone else interrupted the attempt, and/or aborted attempts, where the subject stopped the attempt themselves) using a dichotomous response format, along with the number of each type of attempt. Internal consistency and reliability of the C-SSRS have been widely demonstrated [29,30,31]. The reliability of the interviews was guaranteed through scheduled clinical supervisions. We considered the suicidal ideation scores concerning the past six months, as this timeframe was deemed more appropriate in an emergency clinical context, such as the assessment of suicidal risk in adolescence. This choice also reduced potential inconsistencies in retrospective reporting and temporal overlap with recent ideation.

R-PAS [16]: a performance-based test involving a perceptual task that allows for the detection of the person’s mental functioning and personality structure. The R-PAS method is up-to-date, statistically valid, reliable, and reflects international evidence.

For the present study, suicidal concern was operationalized using a set of R-PAS variables theoretically and empirically associated with suicidal vulnerability in adolescence, rather than as a single diagnostic index. These variables included:

Sum of SR and SI (AnyS): the number of responses that involve the white background as either the primary (SR) or secondary object (SI). One index is related to oppositionality and individualism. The second reflects cognitive effort to solve the task, implying motivation, complex thinking, and creativity.

Human Content (H): refers to the depiction of whole, realistically portrayed humans. It is interpreted as an integrated and comprehensive representation of people. It suggests a cognitively sophisticated and realistic view of oneself and others.

Ordinary Form Quality percent (FQo%): the percentage of conventional and easily perceived responses. A high FQo% demonstrates an accurate perception of the blot’s boundaries and the capacity to interpret the environment accurately, conventionally, and realistically.

Popular responses (P): assigned when an individual sees something commonly associated with the specific blot location.

Form-dominated color responses (FC): These responses involve using colors to identify objects, but identification is primarily based on shape.

Dimensional variables (VFD): each variable is coded when the respondent includes depth and visualizes a third dimension in a response, either by shading or ink contrast (V) or by shape (FD). While the former requires a more complex and sophisticated cognitive process, both involve stepping back, or distancing oneself, which may lead to feelings of insecurity. This distancing may be interpreted more negatively in cases of extreme detachment, such as dissociation, depression, or paranoia.

Color dominance proportion (CFC-FC): the number of responses in which color dominated over shape, minus those in which the opposite was true. It is a rough measure of how cognitive control modulates responses to the environment, especially when an emotional provocation is involved.

Color Blended with Shading or Achromatic Color (CBlend): The number of responses in which color occurs together with shading or achromatic color determinants. In the affective domain, it may involve a tendency for emotionally spontaneous responses to be affected by inconsistency or susceptibility to mixed affective experiences. Noteworthy, according to Zare and colleagues [22], the color-shading variable is the most consistently supported variable across single-marker indicators.

Morbid Content (MOR): Seeing dysphoric, injured, defective, damaged images. It suggests a dysphoric emotional experience or pictures of the self or others as impaired.

LSO-Cmplx: being one of the three components of Complexity and differentiation and integration of response descriptions, it refers to Location, Space, and Object Qualities (LSO), focusing on what areas of the inkblot(s) are involved and whether percepts are seen in meaningful relationships [32].

Coping effectiveness (MC-PPD): obtained by contrasting codes that suggest resources associated with ideational elaboration (movement) and lively responsiveness to the world (chromatic color) to codes that suggest potentially taxing anxiousness (noticing the nuances and subtleties of shading that indicate a sort of an uncomfortable vigilance), disruptive ideation (inanimate or animal movement, which are more primitive types of movement), and dysphoria.

The SC-Comp also contains the Egocentricity Index, which is a weighted sum of responses from the Reflections and Pairs sections [16]. This index was historically intended to assess narcissism. However, given that it showed almost no empirical support as a measure of self-focus, self-esteem, or related concepts [18] and was not included in the R-PAS method, we did not consider it in the analysis.

In 1992, Silberg and Armstrong highlighted the utility of two additional variables from the CS for assessing suicide ideation specifically among adolescents [33].

Given the persistence of these two variables in the R-PAS, they were included as exploratory indicators of cognitive-perceptual disorganization and cognitive inefficiency: M− and WSumCog. These variables were considered theoretically adjacent to suicidality but not redundant with SC-Comp, as they capture broader disruptions in information processing that may contribute to vulnerability in adolescent populations.

M−: scored when human movements occur in responses with an inaccurate perception of the blot’s boundaries. It represents a rough measure of atypical or distorted understanding of people.

WSumCog: the weighted sum of visual and linguistic slipping. It is a measure of disturbed and disordered thought. It can be associated with immature and ineffective thinking and reasoning.

In summary, we selected the SC-Comp from the R-PAS, the most up-to-date version of the S-CON index (CS). We explored its subcomponents, including the CBlend variable, the most validated single marker in the literature [22]. We also explored the CS variables proposed by Silberg and Armstrong [33], which are still present in the R-PAS and were specifically indicated for suicidality in adolescents.

The objective of this study is to examine how these variables may be relevant to suicide ideation in adolescents.

### 2.4. Statistical Analysis

Data were analyzed with RStudio (2026.01.0+392) [34]. Participants with missing data on the primary study instruments (i.e., C-SSRS and R-PAS protocols) were excluded from the analytic sample, as illustrated in the participant flow chart (Figure 1), ensuring that all analyses were conducted on complete cases for the primary measures. Descriptive statistics were performed for sociodemographic, clinical, and R-PAS variables. Interrater reliability was assessed on a random subsample of 30 R-PAS protocols independently coded by a second trained examiner. Intraclass correlation coefficients (ICCs) were calculated following established guidelines [35,36]. Interrater reliability for the R-PAS variables included in the analyses was excellent, with an ICC in the good-to-excellent range (ICC = 0.88, *p* < 0.001, 95% CI [0.77, 0.94]), consistent with the recommended benchmark [37].

To examine potential sex-at-birth-related differences, given the sample imbalance, exploratory comparisons between males and females were conducted. Continuous variables were compared using Mann–Whitney U tests due to non-normal distributions, while categorical variables were analyzed using χ^2^ tests or Fisher’s exact tests.

To examine the multivariate association patterns between R-PAS variables and suicidality, penalized regression models using elastic net regularization were estimated. This approach was chosen to address multicollinearity among R-PAS variables and to evaluate joint multivariate patterns rather than isolated effects of individual indices. Final models were refitted using the value of the regularization parameter (λ) that minimized the cross-validated error, in order to maximize the accuracy and to characterize the full multivariate pattern of associations among R-PAS variables. Selected variables included selected R-PAS standard scores derived from the SC-Comp (AnyS, H, FQo%, P, FC, VFD, CFC-FC, CBlend, MOR, LSO-Cmplx, MC-PPD), and developmentally relevant indices (M− and WSumCog), as well as age and sex at birth. Sociodemographic (SES) and clinical factors (any psychotic disorder, any depressive disorder, any anxiety disorder, any eating disorder) were also included in the candidate set but did not survive regularization. All variables were standardized prior to analysis to enable direct comparison of regression coefficients, using centering and scaling procedures applied before model estimation.

Two outcome variables derived from the C-SSRS were examined: (a) suicidal ideation severity in the six months prior to assessment, treated as a continuous variable (0–5), and (b) suicidal behavior, treated as a dichotomous variable reflecting the presence of any actual, interrupted, or aborted suicide attempt across the lifetime. The distribution of suicidal ideation scores showed mild negative skewness (−0.31), indicating a slight concentration of higher severity values, but no substantial deviation from symmetry.

A grid of elastic net mixing parameters (α = 0.1–1.0) was evaluated using 10-fold cross-validation. For the suicidal ideation severity model (continuous outcome), model selection was based on minimizing cross-validated mean squared error (MSE), with R^2^ and root mean squared error (RMSE) reported for descriptive purposes. For the suicidal behavior model (binary outcome), model selection was based on maximizing the cross-validated AUC, with additional evaluation of in-sample AUC and Brier score for calibration. Final models were refit using the optimal α value and the corresponding λ that minimized the cross-validated error.

To further evaluate the stability of model estimates and variable selection, we examined sensitivity to parameter selection and resampling variability. In addition to λ.min, we inspected λ.1se solutions to assess the robustness of selected predictors under stronger penalization. Moreover, bootstrap stability analyses were conducted using 500 resampled datasets. For each resample, the elastic net model was re-estimated using the optimal α values and the frequency with which each variable (non-zero coefficient) was recorded. This procedure provided an estimate of the stability of variable selection across resampled datasets and allowed for identification of variables that were consistently selected versus those sensitive to sampling variability.

Together, cross-validation across folds and bootstrap-based stability selection were used to reduce dependence on a single data split and to characterize the robustness of the identified multivariate patterns. Penalized regression analyses were conducted on 153 adolescents.

## 3. Results

### 3.1. Descriptives

The study sample comprised 153 adolescents, with a mean age of 15.1 ± 1.44 years and a predominantly female composition. Table 1 summarizes sociodemographic characteristics, diagnoses, medications, and suicidality indicators of the sample, and Table 2 reports descriptive statistics for the R-PAS variables entered as candidate variables in the penalized regression models.

Given the marked predominance of female participants, exploratory analyses were conducted to examine potential sex-at-birth differences in clinical and psychological variables (Table S2). No significant differences emerged between males and females in suicidal ideation severity (*p* = 0.323), suicidal behavior indicators (all *p* > 0.14), or R-PAS variables (all *p* > 0.10). Similarly, no differences were observed in age or SES. Diagnostic distributions were broadly comparable across sexes, although depressive disorders were more frequent among females (*p* = 0.40) as well as eating disorders (*p* = 0.012). Overall, these findings suggest that the observed multivariate patterns are unlikely to be driven by sex-related differences within the sample.

### 3.2. Multivariate Associations with Suicidal Ideation Severity

The elastic net model for suicidal ideation severity (past 6 months) identified an optimal mixing parameter of α = 0.90, indicating a more LASSO-like solution. The model retained three R-PAS variables with non-zero coefficients. Higher scores on LSO-Cmplx, MOR, and VFD were associated with greater severity of suicidal ideation. No other R-PAS variables, sociodemographic, or clinical variables were retained after penalization (Table 3).

Model performance indicated modest explanatory capacity, consistent with the multifactorial nature of suicidal ideation. The in-sample coefficient of determination was R^2^ = 0.09, with a RMSE of 1.79. Although explanatory performance was modest, the model identified a consistent multivariate pattern of associations that was stable across resampling procedures.

### 3.3. Multivariate Associations with Suicidal Behavior

For suicidal behavior (presence vs. absence of any actual, interrupted, or aborted attempt), the elastic net model selected a balanced parameter of α = 0.40. The final model indicated that higher levels of MOR and LSO-Cmplx were associated with a higher likelihood of having engaged in suicidal behavior (Table 4). Model discrimination was acceptable. The cross-validated AUC for the optimal model was 0.66, and the in-sample AUC was 0.65. Calibration was assessed using the Brier score, which was 0.24, indicating only moderate calibration, consistent with the overall modest discriminative performance of the model.

Across both elastic net models, MOR and LSO-Cmplx emerged as consistent multivariate correlates of suicidality, being retained in both ideation severity and suicidal behavior models. VFD was not retained in the suicidal behavior model. Similarly, other R-PAS variables included in the models were not retained after regularization, suggesting limited multivariate association when considered jointly with the retained variables.

To further examine the robustness of the identified multivariate patterns, bootstrap stability analyses were conducted, indicating that for suicidal ideation, MOR and LSO-Cmplx emerged as the most consistently selected variables across bootstrap resampling, whereas VFD showed moderate selection stability. For suicidal behavior, MOR showed the highest selection stability, followed by LSO-Cmplx.

## 4. Discussion

The present study examined multivariate patterns of R-PAS variables associated with suicidal ideation severity and suicidal behavior in a clinical sample of help-seeking adolescents. Using elastic net regularization, we did not identify strong standalone predictors of suicidal ideation or behavior but rather pointed to a partially stable multivariate pattern of associations with suicidal risk features. Across both models, MOR emerged as the most consistent multivariate correlate of suicidality. Higher MOR scores have been associated with a tendency to perceive stimuli as damaged, dead, or dysphoric, which may be consistent with prior literature describing an inner world populated by pessimistic, self-critical, or life-denying ideational themes. This finding aligns with a substantial body of literature linking dysphoric mood, negative self-concept, and death-oriented thinking to suicidal concerns in adolescents [38,39,40].

Morbid responses could be associated with both internal emotional states and the individual’s lived environment, reflecting experiences of loss, shame, or bodily distress. Prior work has suggested that adolescents with problematic bodily experiences or negative self-representations show more concerning attitudes toward death and reduced investment in life [41]. Within a performance-based framework, MOR reflects the presence of dysphoric and morbid ideational content in perceptual responses, without implying suicidal intent.

Furthermore, MOR responses may reflect content that is experientially congruent with negative affective states [16]. The R-PAS authors suggest that “Like many other Thematic Codes, MOR is subject to impression management” [16] (pp. 345–346). Previous literature highlighted that adolescents with lower emotional competence may be less likely to seek help from peers or family, but remain willing to engage with mental health professionals [42], particularly when supported by encouragement from family or friends [43]. Even if the present data do not allow for inferences about communicative intent or help-seeking processes during assessment, describing percepts as damaged, sad, or dead may be explored as an implicit communicative strategy through which adolescents convey distress and seek recognition from the examiner.

LSO-Cmplx was retained in both elastic net models, indicating that higher levels of perceptual organization and complexity were associated with both greater ideation severity and suicidal behavior. LSO-Cmplx has been interpreted as the ability to differentiate and synthesize environmental features, and dorsal attention, an active form of voluntary attention directed toward the environment [44]. Elevated LSO-Cmplx scores have been interpreted as reflecting increased cognitive effort and perceptual organization, which may be associated with heightened monitoring or evaluative processing in certain contexts [45].

Higher LSO-Cmplx scores indicate greater effort in scanning the environment, and a persistent cognitive fatigue in life experiences. This pattern is broadly consistent with prior literature linking higher spatial-cognitive-perceptual complexity with increased evaluative processing and self-referential cognition (e.g., anxiety, eating disorders, self-harm, and suicidal ideation) [46,47,48,49,50]. In this sense, LSO-Cmplx scores may have increased due to the persistent over-vigilance, rumination, evaluation, and self-monitoring, rather than suicidal ideation, motivational, or symbolic content, although no direct inference regarding maladaptive perfectionism, typically associated with this functioning, can be made from the present data.

Concerning VFD, it contributed only to the ideation severity model, although with smaller effect sizes. VFD has been associated with the tendency toward vigilance combined with avoidance. This index has been interpreted as reflecting forms of perceptual distancing from emotionally or perceptually overwhelming stimuli or attentional disengagement, which may be associated with coping responses to distress. Previous research has shown that avoidance-based coping strategies are positively associated with suicidal risk in adolescents, regardless of sex at birth [51]. In the present study, VFD co-occurred with MOR and LSO-Cmplx, suggesting a potential pattern of association between perceptual avoidance indicators and other cognitive–affective variables. These findings should also be considered within the broader debate surrounding performance-based assessment methods, particularly regarding the extent to which R-PAS variables capture stable psychological traits versus context-dependent performance processes.

Taken together, the retained variables describe a set of weak-to-moderate multivariate associations involving dysphoric ideational content, greater effort in scanning the environment, and avoidant-related indicators may show partial overlap with constructs discussed in contemporary models of adolescent suicidality, including the co-occurrence of emotional fatigue and cognitive overload [52]. The magnitude of the observed associations was modest, consistent with the low explanatory and discriminative performance of the models, and does not support interpretation at the level of individual clinical prediction or profile characterization.

In the context of suicide behaviors, the primary concern is the occurrence of false negatives. Given the well-documented fragility of predictive indices in suicide research more broadly, we would like to consider the possibility that adolescents with R-PAS profiles in which the SC-Comp is below the threshold, MOR, LSO, and VFD may alert clinicians concerning processes the client may not be fully aware of.

Although this study was not designed to test any theoretical models directly, our results are partially compatible with the most widely validated theoretical approaches to suicidal behaviors and ideation. The presence of dysphoric and negative ideational content (i.e., MOR) and patterns of perceptual disengagement (VFD) may show conceptual overlap with constructs such as perceived burdensomeness and reduced connectedness described in the interpersonal theory of suicide [53,54]. Similarly, the combination of negative ideational content (MOR) and increased demands on cognitive organization (LSO-Cmplx) may align with stress–diathesis frameworks [55,56], in which stable vulnerabilities interact with stress-related processes. Finally, these findings are partially coherent with the Integrated Motivational–Volitional model [57], emphasizing persistent negative cognition, cognitive load, and maladaptive coping and their role in the transition from suicidal ideation to behaviors.

### Constraints on Generality

This study presents some limitations. First, although sex at birth was included as a covariate in all multivariate models, the sample was predominantly female, limiting the generalizability of the results, even though comparison analyses showed no statistically significant differences by sex at birth. This reflects differences in help-seeking tendencies, with females seeming to have more confidence in mental health professionals while young males ask for help from friends and parents and prefer self-reliance to cope with mental issues [58]. However, this sex-at-birth distribution reflects the clinical composition of help-seeking adolescent populations, where females are overrepresented in presentations involving internalizing symptoms and suicidality. It may also be due to the higher mortality rate among males than females [59]. Second, the group consisted solely of individuals referred to specialized mental health services and diagnosed with severe neuropsychiatric disorders and not healthy controls, thus limiting the extent to which the findings can be generalized to adolescents in the general population or those not seeking care. Third, despite the use of penalized regression techniques that handle multicollinearity, results should be interpreted as components of multivariate patterns associated with suicidal ideation severity and suicidal behavior, rather than as isolated or causal risk factors. Moreover, the operationalization of suicidal ideation as a continuous variable and the dichotomization of suicidal behavior may have introduced measurement constraints, potentially reducing sensitivity to gradations in severity and heterogeneity. Furthermore, the participants were self-selected, and all lived in Italy, having consented to participate in this study from a larger cohort that referred to our mental health services. Moreover, although cross-validation and bootstrap procedures were used to reduce overfitting, replication in independent samples is needed to establish the robustness of these associations. In fact, the absence of external validation limits the ability to assess generalizability. Future studies should replicate these findings in independent clinical cohorts and across different cultural contexts.

Finally, the cross-sectional design precludes drawing temporal or causal relationships between R-PAS variables and suicidal ideation or behavior. As such, the identified associations should not be interpreted as reflecting underlying mechanisms or risk trajectories. Longitudinal studies are needed to determine whether the identified R-PAS patterns prospectively predict changes in suicidal ideation or the occurrence of suicidal behaviors over time. Future research should also examine whether additional R-PAS variables not included in the present models contribute to suicidal risk, and whether integrating R-PAS data with other clinical, environmental, or biological measures improves risk stratification in adolescent populations.

## 5. Conclusions

Crucially, these findings do not support the use of the SC-Comp as a standalone indicator of suicidal risk in adolescents. Nevertheless, the R-PAS is commonly used for the general assessment of personality functioning. In clinical contexts where the R-PAS is already administered, certain response patterns may contribute additional descriptive information about cognitive–affective functioning, but may also help in detecting risky cognitive styles in adolescents. This could prompt the clinician to monitor possible suicidal thoughts. Furthermore, the clinical value of the R-PAS lies in elucidating the underlying psychological processes that shape how adolescents experience, regulate and communicate distress. Although performance-based assessment methods such as the R-PAS offer clinically rich information, they also present practical limitations that may constrain their routine use in acute or high-throughput clinical settings (e.g., time requirements for administration and coding, the need for trained, reliable raters, and implementation within standard practice). As a result, while the present findings support meaningful multivariate associations, their direct translation into routine clinical decision-making should be approached with caution and within the constraints of ecological validity and feasibility.

The present findings suggest that changes in morbid ideational content, cognitive complexity, or perceptual rigidity across assessments may reflect changes in psychological processes that could be relevant for clinical formulation, even when self-reported suicidal ideation remains stable, although this interpretation requires further validation and the present findings do not support the use of R-PAS variables as standalone tools for suicide risk prediction or clinical decision-making.

Integrating R-PAS with other instruments, such as self-report questionnaires [60], in the framework of structured risk assessment may provide supplementary information on adolescents whose distress may be maintained by persistent rumination, effort in monitoring, or emotional disengagement, which can be considered alongside established clinical assessment methods to inform intervention proposals. Importantly, these findings should be interpreted in light of key methodological constraints. The cross-sectional design precludes causal inferences, and the modest explanatory power of the models suggests that substantial variance remains unexplained. Accordingly, the observed associations should be considered exploratory and hypothesis-generating. Further longitudinal and independent replication studies are needed to clarify the robustness and interpretive significance of these relationships.

## Figures and Tables

**Table 1:** Sociodemographic characteristics of the total sample.

Variable		
Age, M (SD)	15.1	1.44
Sex at birth (female), *n* (%)	131	85.62
Ethnicity, *n* (%)		
Caucasian	123	80.39
Asian	4	2.61
African	8	5.29
South America	7	4.55
Mix	11	6.91
Familiarity with psychiatric disorders (yes), *n* (%)	72	47.06
Social relationships, *n* (%)		
Unknown	2	1.30
Withdrawal	22	14.38
Poor	76	49.67
Good	53	34.64
SES, M (SD)	34.12	14.20
Unknown	4	2.60
Low (8–19)	20	13.07
Middle-low (20–29)	38	24.83
Middle (30–39)	28	18.30
Middle-high (40–54)	31	20.26
High (55–66)	14	9.15
Academic performance, *n* (%)		
Unknown	2	1.31
School withdrawal	24	15.69
Poor	35	22.88
Sufficient	59	38.56
Good	22	14.38
Excellent	11	7.19
Risk Conducts (yes), *n* (%)	85	55.56
Diagnoses, *n* (%)		
Psychotic disorders ^a^	21	13.73
Depressive disorders ^b^	91	59.48
Anxiety disorders ^c^	63	41.18
Restrictive eating disorders ^d^	40	26.14
Medications (yes), *n* (%)	70	45.75
Antipsychotics	28	18.30
Antidepressants	33	21.57
Benzodiazepines	46	30.07
Mood stabilizers	7	4.58
Suicidal ideation severity (past 6 months), M (SD)	2.78	1.90
No suicidal ideation	30	19.61
C-SSRS SI1, *n* (%)	19	12.42
C-SSRS SI2, *n* (%)	12	7.84
C-SSRS SI3, *n* (%)	22	14.38
C-SSRS SI4, *n* (%)	32	20.92
C-SSRS SI5, *n* (%)	38	24.84
Presence of any suicidal behavior, *n* (%)	74	48.37
C-SSRS SB1, *n* (%)	47	30.72
C-SSRS SB2, *n* (%)	17	11.11
C-SSRS SB3, *n* (%)	38	24.84

Abbreviations: C-SSRS SI1 = wish to be dead; C-SSRS SI2 = non-specific active suicidal thoughts; C-SSRS SI3 = active suicidal ideation with any method but without a specific plan or the intent to act; C-SSRS SI4 = active suicidal ideation with some intent to act but without a specific plan; C-SSRS SI5 = active suicidal ideation with a detailed plan and intention to die; C-SSRS SB1 = actual attempts; C-SSRS SB2 = interrupted attempts; C-SSRS SB3 = and/or aborted attempts; SES = socioeconomic status. Note. ^a^ Psychosis, Attenuated Psychotic Syndrome, Unspecified Schizophrenia Spectrum and Other Psychotic Disorder; ^b^ Major Depressive Disorder, Dysthymia, Disruptive Mood Dysregulation Disorder; ^c^ Generalized Anxiety Disorder, Separation Anxiety; ^d^ Anorexia Nervosa, Atypical Anorexia Nervosa, Bulimia Nervosa, Binge-eating.

**Table 2:** Descriptive statistics for R-PAS variables (*n* = 153).

Variable	M	SD
AnyS	102.04	15.88
H	104.07	12.25
FQo%	92.89	15.50
P	94.06	12.77
FC	97.92	11.48
CFC-FC	103.93	15.44
VFD	100.76	11.30
CBlend	102.48	11.51
MOR	107.96	14.88
LSO-Cmplx	99.78	15.94
MC-PPD	101.59	12.91
M−	108.54	14.64
WSumCog	102.50	15.83

**Table 3:** Elastic net-selected R-PAS variables associated with suicidal ideation severity.

Variable (Standardized)	Coefficient	Selection Frequency
LSO-Cmplx	0.249	0.55
MOR	0.175	0.48
VFD	0.043	0.24

Note. Selection frequency: >0.60 stable, 0.40–0.60 moderate, <0.40 weak/unstable.

**Table 4:** Elastic net-selected R-PAS variables associated with the presence of suicidal behavior.

Variable (Standardized)	Coefficient	OR	Selection Frequency
MOR	0.090	1.09	0.81
LSO-Cmplx	0.035	1.04	0.63

Note. Selection frequency: >0.60 stable, 0.40–0.60 moderate, <0.40 weak/unstable.

## Data Availability

The data supporting the findings of this study are openly available in Zenodo at https://zenodo.org/records/10528510 [24]. The data are not published elsewhere, and the data from this study have not been reported in any other publications.

## Supplementary Materials

The following supporting information can be downloaded at: https://www.mdpi.com/article/10.3390/brainsci16050519/s1, Table S1: The RECORD statement—a checklist of items, extended from the STROBE statement, that should be reported in observational studies using routinely collected health data [61]. Table S2: Sex-at-birth differences in sociodemographic, clinical, and R-PAS variables.

## Abbreviations

The following abbreviations are used in this manuscript:

AnyS	Sum of SR and SI
CBlend	Color Blended with Shading or Achromatic Color
CF	Color-dominated color responses
CFC-FC	Color dominance proportion
CS	Comprehensive System
C-SSRS	Columbia-Suicide Severity Rating Scale
FC	Form-dominated color responses
FD	Third dimension because of the shape
FQo%	Ordinary Form Quality percent
H	Human Content
ICC	Intraclass correlation coefficient
LSO-Cmplx	Location, Space, and Object Qualities-Complexity
M−	Human movements in FQ-responses
MC-PPD	Coping effectiveness
MOR	Morbid Content
NSSI	Non suicidal self-injury
P	Popular responses
R-PAS	Rorschach Performance Assessment System
SC-Comp	Suicide Concern Composite
S-CON	Suicide Constellation
SES	Socioeconomic status
SI	Responses that involve the white background as the secondary object
SR	Responses that involve the white background as the primary object
V	Third dimension because of the shading or contrasts in the ink
VFD	Dimensional variables
WAIS-IV	Wechsler Adult Intelligence Scale—Fourth Edition
WISC-IV	Wechsler Intelligence Scale for Children—Fourth Edition
WSumCog	Weighted sum of visual and linguistic slipping
